# Supplementary material for: Curcumin Stereoisomer, Cis-Trans Curcumin, as a Novel Ligand to A1 and A3 Adenosine Receptors
Source: Pharmaceuticals (Basel). 2023 Jun 22;16(7):917. doi: 10.3390/ph16070917 (PMC10385834; doi:10.3390/ph16070917)
Supplement: Supplementary file 1 [file pharmaceuticals-16-00917-s001.zip › pharmaceuticals-2330233-supplementary.pdf]

Methods

Cells were seeded at 50,000 cells per well in a 96-well plate and allowed to incubate overnight. After running our first twenty cyclic AMP assays, we reduced this seeding density down to 35,000 cells per well to ensure that the cells would still be in log phase when they were treated the next day (i.e., to ensure that the cells would not reach confluence overnight). After the overnight incubation, cells were treated with test compounds or control solutions and allowed to incubate for 2h. To test for agonistic activity, 50 μM forskolin was used as a control stimulator of adenylate cyclase, and CTCUR was added to see if it would activate the G_i-linked receptors and mitigate the effect of forskolin. Forskolin was added at the 1h 45 min mark, such that cells were only incubated with the forskolin for 15 min. After incubation was complete, the lysing of the cells and the remaining parts of the assay were performed according to the manufacturer's protocol (ab138880 cAMP Direct Immunoassay Kit, Abcam, Cambridge, UK). The plate was read with a Synergy H1 microplate reader (BioTek, Winooski, VT, USA).

Results

Immunoassay results support the hypothesis that CTCUR binds to A_1AR. Forskolin + 10 μM CTCUR produced a 27.5 nM reduction in cAMP concentration compared to the forskolin control (Supplementary Figure 1). Since A_1AR is G_i-linked, the observed reduction in cAMP concentration in response to CTCUR suggests that CTCUR acts as an agonist at A_1AR. There was a 23.84 nM reduction in cAMP concentration in response to the forskolin + 10 μM CTCUR treatment compared to the forskolin control (Supplementary Figure 2). Although not statistically significant, this result is consistent with the receptor activation results for A_1AR, and it thus supports the hypothesis that CTCUR acts as an agonist of ARs.

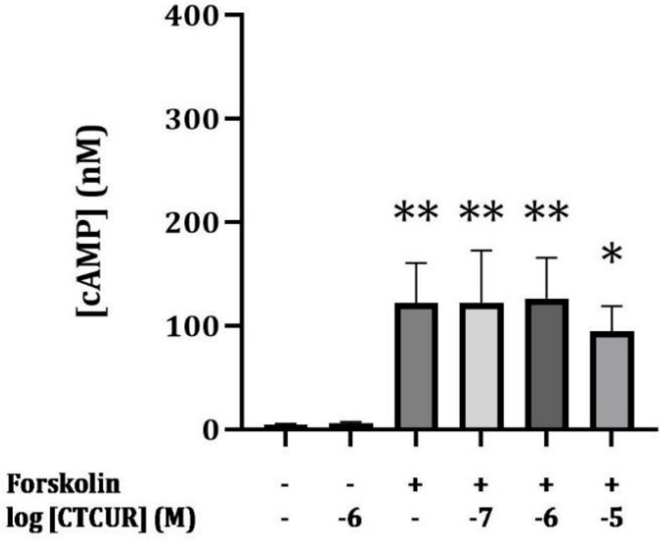

Figure S1. Test for agonistic activity of CTCUR at A_1AR (B, n=3). Forskolin was administered at 50 μM to directly activate adenylate cyclase and thereby elevate cAMP levels. Since A_1AR is G_i-linked, reduction in cAMP levels indicates agonism of the receptor. Asterisks indicate significant difference from both the media control (-, -) and the CTCUR control (-, -6). * indicates $0.01 < p \leq 0.05$, ** indicates $p \leq 0.01$. Bars indicate standard deviation.

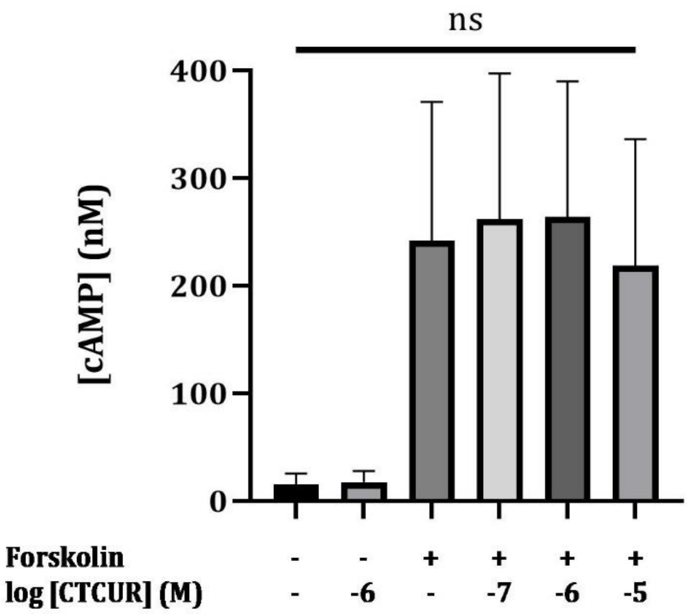

Figure S2. Test for agonistic activity of CTCUR at A_3AR (B, n=3). Forskolin was administered at 50 μM to directly activate adenylate cyclase and thereby elevate cAMP levels. Since A_3AR is G_i-linked, reduction in cAMP levels indicates agonism of the receptor. Bars indicate standard deviation.

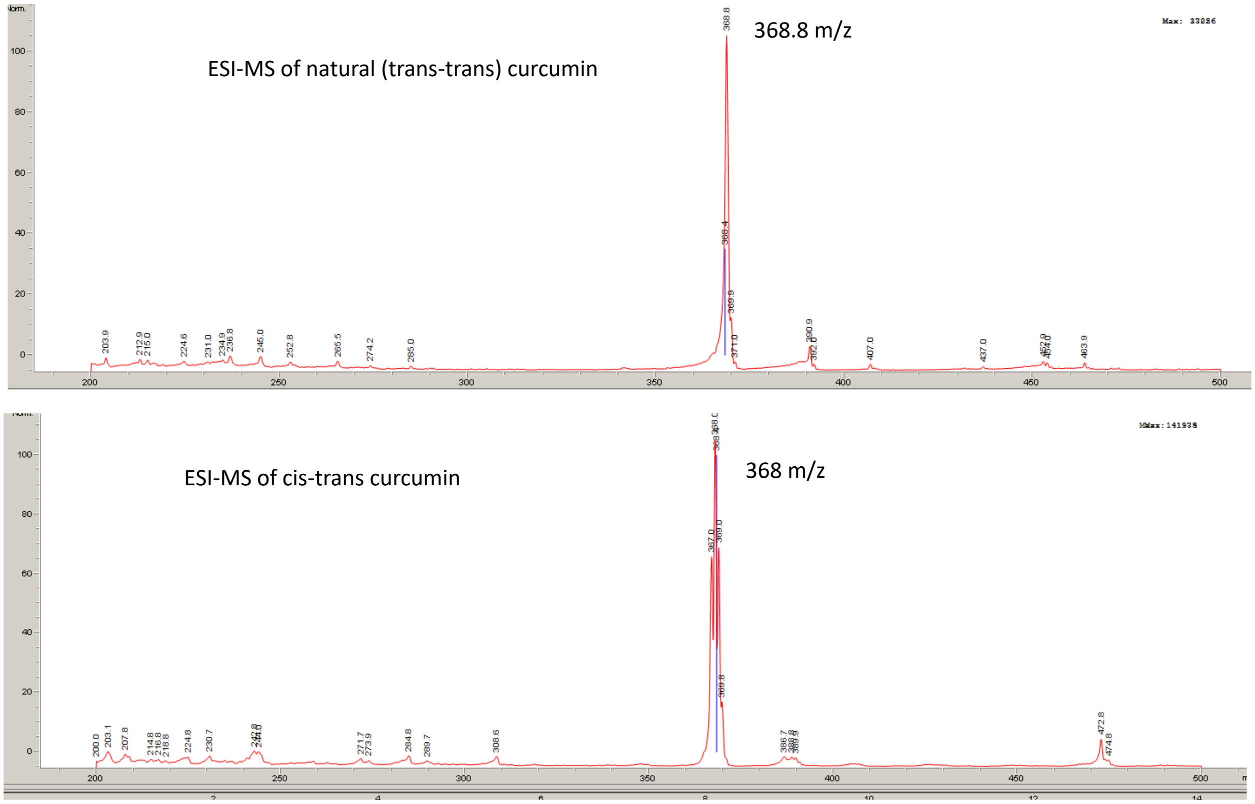

Figure S3. ESI-MS spectra of natural (*trans-trans*) and synthesized *cis-trans* curcumins.
